# Alteration in glucose homeostasis and persistence of the pancreatic clock in aged *mPer2*^*Luc*^ mice

**DOI:** 10.1038/s41598-018-30225-y

**Published:** 2018-08-03

**Authors:** Zuzana Novosadová, Lenka Polidarová, Martin Sládek, Alena Sumová

**Affiliations:** 1 Department of Neurohumoral Regulations, Institute of Physiology, the Czech Academy of Sciences, Prague, Czech Republic; 2 Faculty of Sciences, Charles University, Prague, Czech Republic

## Abstract

The physiological function of the pancreas is controlled by the circadian clock. The aim of this study was to determine whether aging-induced changes in glucose homeostasis affect properties of the circadian clock in the pancreas and/or its sensitivity to disturbances in environmental lighting conditions. *mPer2*^*Luc*^ mice aged 24–26 months developed hyperinsulinemic hypoglycaemia, which was likely due to the *Pclo*-mediated insulin hyper-secretion and *Slc2a2*-mediated glucose transport impairment in the pancreas, and due to the alterations in *Pp1r3c*-related glycogen storage and *Sgk1*-related glucose transport in the liver. In the pancreatic tissue, aging affected clock gene expression only marginally, it upregulated *Bmal1* and downregulated Clock expression. Whereas aging significantly impaired the circadian clock in lung explants, which were used as a control tissue, the properties of the pancreatic clock *in vitro* were not affected. The data suggest a non-circadian role of *Bmal1* in changes of pancreatic function that occur during aging. Additionally, the pancreatic clock was more sensitive to exposure of animals to constant light conditions. These findings provide an explanation for the previously demonstrated relationship between disturbances in the circadian system and disordered glucose homeostasis, including diabetes mellitus type 2, in subjects exposed to long-term shift work.

## Introduction

The pancreas, a part of the gastrointestinal tract essential for glucose homeostasis, changes during the lifespan and aging affects the morphology of its exocrine as well as endocrine parts. However, the age-dependent changes in morphology of the exocrine pancreas were not associated with changes in its secretory capacity^1,2^. The endocrine pancreas also undergoes morphological changes with aging^3–5^, however, the reported effects of aging on insulin levels in blood have not been consistent (reviewed in^6^). Nevertheless, in all studied models (humans, rats and mice) aging is associated with impaired glucose tolerance, insulin resistance and altered pancreatic secretory capacity. Whether and how these changes might be caused by aging *per se*, or by other factors, such as body composition, diet or level of physical activity, is still not known^7^.

Pancreatic function is under control of the endogenous circadian system, and the circadian clocks residing in the pancreas are involved in optimizing pancreatic function in accordance with changing demands during the day^8,9^. The pancreas harbours the circadian clocks in its exocrine^10^ as well as endocrine^11^ parts; the latter has been extensively studied mainly because of its role in regulation of insulin release from β-cells forming the islets of Langerhans^12^. The circadian system is organized in the body hierarchically such that peripheral clocks in various tissues, including the pancreas, are synchronized by the central clock in the suprachiasmatic nuclei (SCN) of the hypothalamus^13^. The SCN receives information about the external light/dark (LD) cycle via a connection with the retina and sends multiple signals to the periphery (reviewed in^14^) adjusting the autonomous rhythmicity of the pancreatic clock to rhythmicity of external environment. The generation of the rhythmic signal is based on the core molecular clock mechanism formed by transcriptional-translational feedback loops that drive circadian expression of clock genes, namely, *Per1/Per2*, *Cry1/Cry2*, *Clock/Npas2*, *Bmal1/Bmal2*, *Nr1d1/Nr1d2 (Rev-erbα/Rev-erbβ*) (for review see^15^). Most components of the core clock mechanism, also demonstrated in the pancreas^9^, are rhythmically expressed and serve thereby as downstream transcription factors rhythmically switching on and off expression of a great array of tissue-specific, clock-controlled genes. The functional significance of the clock in the pancreas was confirmed in mice with clock gene deletions *(Bmal1*^*−/−*^*)* or mutation (*Clock*^*Δ19/Δ19*^) that also led to a reduction in the percentage of large islets of Langerhans^11,16^. Additionally, mice with disruption of *Bmal1* selectively in the β-cells exhibited altered glucose tolerance *in vivo* and *in vitro*^11^. Moreover, frequent disruption of the circadian system, e.g., due to night shifts, has been associated with development of diabetes mellitus type II (T2DM) in humans^17,18^.

The impact of aging on the circadian clocks has not been resolved yet. For the central SCN clock, the accumulated data favour the hypothesis that the core oscillatory mechanism is not impaired^19^ but the aged SCN loses the ability to drive the output rhythms at the level of the SCN neuronal activity^20^ or at the systemic behavioural level^19,21,22^. It is not known whether the weakened signals from the SCN to the periphery in aging may impair the internal synchrony among peripheral clocks within the body, although such a mechanism seems plausible from recent evidence for attenuated sympathetic tonus in aged animals^23^. Studies on aging of the peripheral clocks also have not been conclusive. In a study using transgenic *Per1-luc* rats, a tissue-specific effect of aging was reported *in vitro*^24^. In transgenic *mPer2*^*Luc*^ mice, aging changed the dynamics of re-entrainment of bioluminescence rhythms to a shift in LD cycle also in a tissue-dependent manner^25^. Recently, a study on the effect of aging on the circadian transcriptome revealed changes in cyclic protein acetylation in the liver^26^. Because none of the previous studies addressed aging of the clock in the pancreas, we used transgenic *mPer2*^*Luc*^ mice with age-induced impairment in glucose homeostasis to reveal the effect of aging on properties of the circadian clocks in the pancreas. We used lungs as a control tissue not involved in glucose homeostasis. Moreover, we tested the effect of aging on these clocks in conditions where, in addition to aging, the output of the SCN was disrupted via exposure of mice to a disturbance in the environmental LD cycle. These experiments aimed to answer the questions whether the peripheral clock in the pancreas changed with age and whether the rhythmic environment may contribute to the functioning of the pancreatic circadian clock in the elderly.

## Results

### Aging affects pancreatic function and glucose homeostasis mechanisms *in vivo*

**Figure 1:**
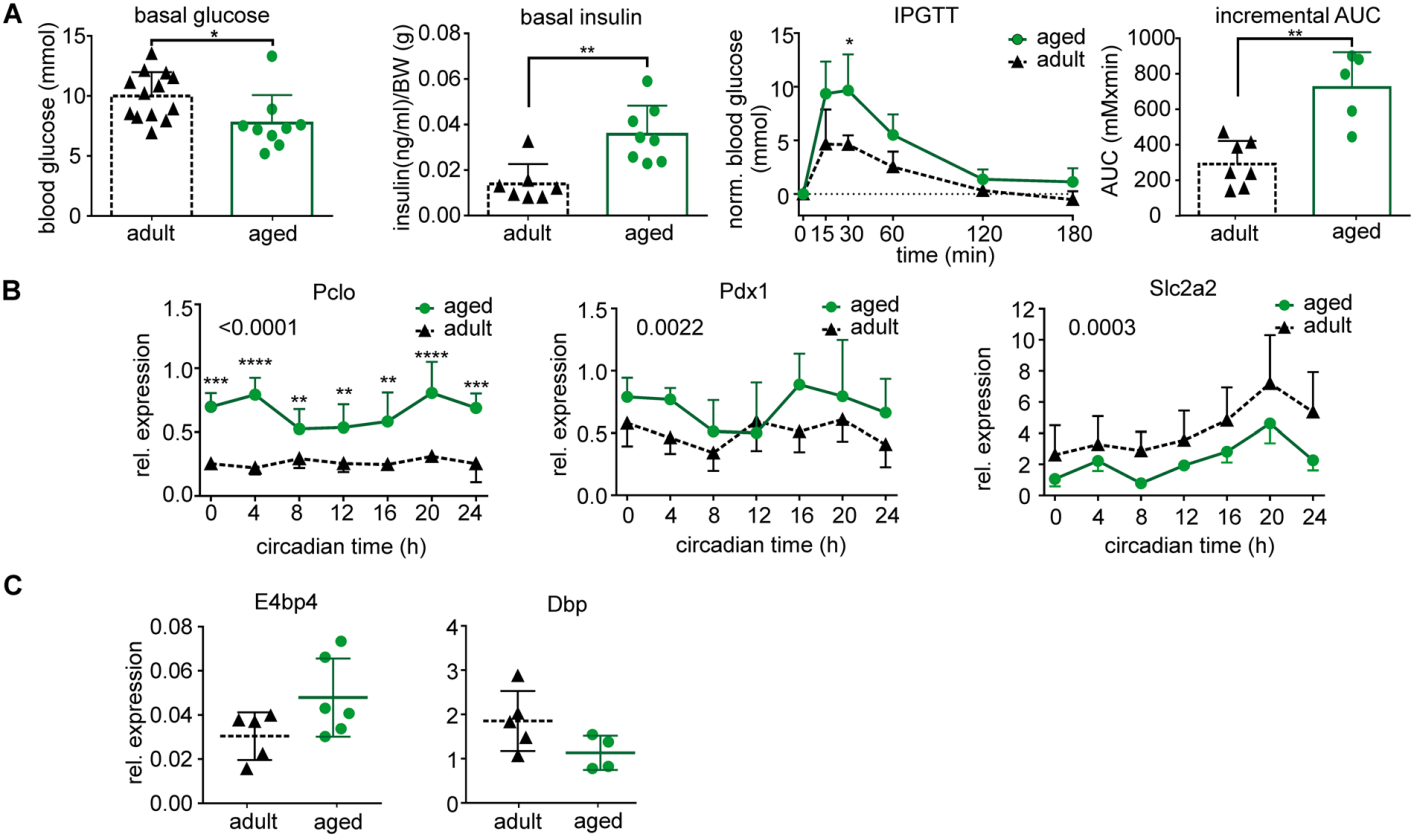
Glucose homeostasis and pancreatic gene expression in adult (black triangles) and aged (green circles) animals maintained under standard light/dark conditions. (**A**) Basal plasma glucose and insulin levels for each group are depicted as individual levels as well as the mean ± SD. Basal insulin levels are expressed relative to body weight (BW). Glucose tolerance test (IPGTT) was performed by measuring plasma glucose levels 15, 30, 60, 120 and 180 min after intraperitoneal injection of glucose (levels normalized to basal glucose levels), and values of the incremental area under the curve (AUC) were calculated. (**B**) Daily profiles in relative expression of *Pclo*, *Pdx1* and *Slc2a2* were detected by RT qPCR in samples of pancreas collected from adult (black triangles and dashed line) and aged (green circles and full line) animals every 4 h during the 24 h profile. Animals were sacrificed in darkness; circadian time 0 corresponds to lights on of the previous LD12:12 regime. At each time point, 5 (occasionally 4) animals were sacrificed and data are expressed as the mean ± SD. The results of a post hoc analysis from a 2-way ANOVA comparison between expression levels in adult and aged animals are depicted. (**C**) The expression of *E4bp4* and *Dbp* in the pancreas of adult (black) and aged (green) animals was detected at circadian times 0 and 12, respectively, i.e., at the time of their maximal levels; for each group, data are depicted as individual levels as well as the mean ± SD. *P < 0.05; **P < 0.01; ***P < 0.001; ****P < 0.0001.

In aged *mPer2*^*Luc*^ mice, basal plasma glucose levels (Fig. 1A) were lower (P = 0.0117) and insulin levels were higher (P = 0.0033) than in adults. Additionally, the IPGTT revealed that glucose levels 30 min after glucose administration, as well as the incremental area under the curve (AUC), were significantly higher in aged animals (P < 0.0001 and P = 0.0045, respectively). In the pancreatic tissue, aging had a significant effect on gene expression (Fig. 1B). It upregulated *piccolo (Pclo)* mRNA levels (P < 0.0001) throughout the 24 h cycle. Expression of *pancreatic and duodenal homeobox 1 (Pdx1)* gene was also upregulated significantly by aging (P = 0.0022), although post hoc analysis did not find significant differences at individual time points. In contrast, expression profile of *solute carrier family 2 member 2 (Slc2a2)* gene for glucose transporter GLUT2 was downregulated by aging (P = 0.0003), although post hoc analysis did not detect significant differences at individual time points. Aging did not significantly affect maximal expression levels of transcription factor *E4bp4* (P = 0.1255) and *D-box binding protein* (*Dbp*) (P = 0.1111) in the pancreas.

### Aging affects amplitudes of Bmal1 and Clock rhythms in a gene-specific and peripheral tissue-specific manners ***in vivo***

**Figure 2:**
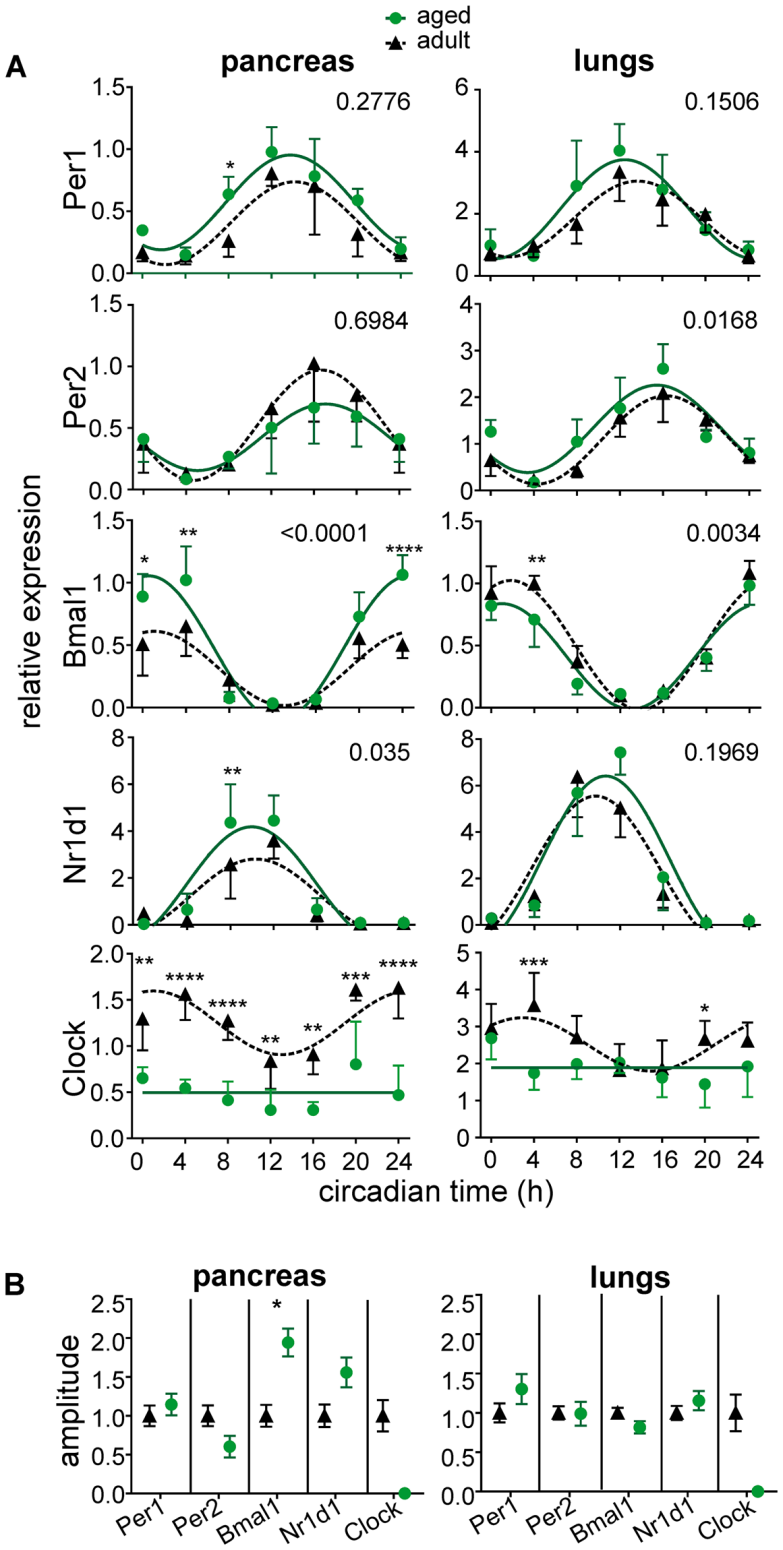
Effect of aging on expression of clock genes in the pancreas and lungs *in vivo*. (**A**) Daily profiles of relative expression of *Per1*, *Per2*, *Bmal1*, *Nr1d1* and *Clock* were detected by RT qPCR in samples of adult (black triangles, dashed line) and aged (green circles, solid line) animals. The tissue samples were collected every 4 h during the 24 h profile. Animals were sacrificed in darkness; circadian time 0 corresponds to lights on of the previous LD12:12 regime. At each time point, 5 (occasionally 4) animals were sacrificed and data are expressed as the mean ± SD. For each gene and tissue, the results of a 2-way ANOVA comparison between the profiles of adult and aged animals are depicted as the P value. The data were fitted with cosine curves. (**B**) Comparison of amplitudes of the rhythmic gene expression in adult (black triangles, dashed line) and aged (green circles, solid line) animals depicted in A) detected by cosine analysis. Data are expressed at mean ± SEM. *P < 0.05; **P < 0.01; ****P < 0.0001.

**Figure 3:**
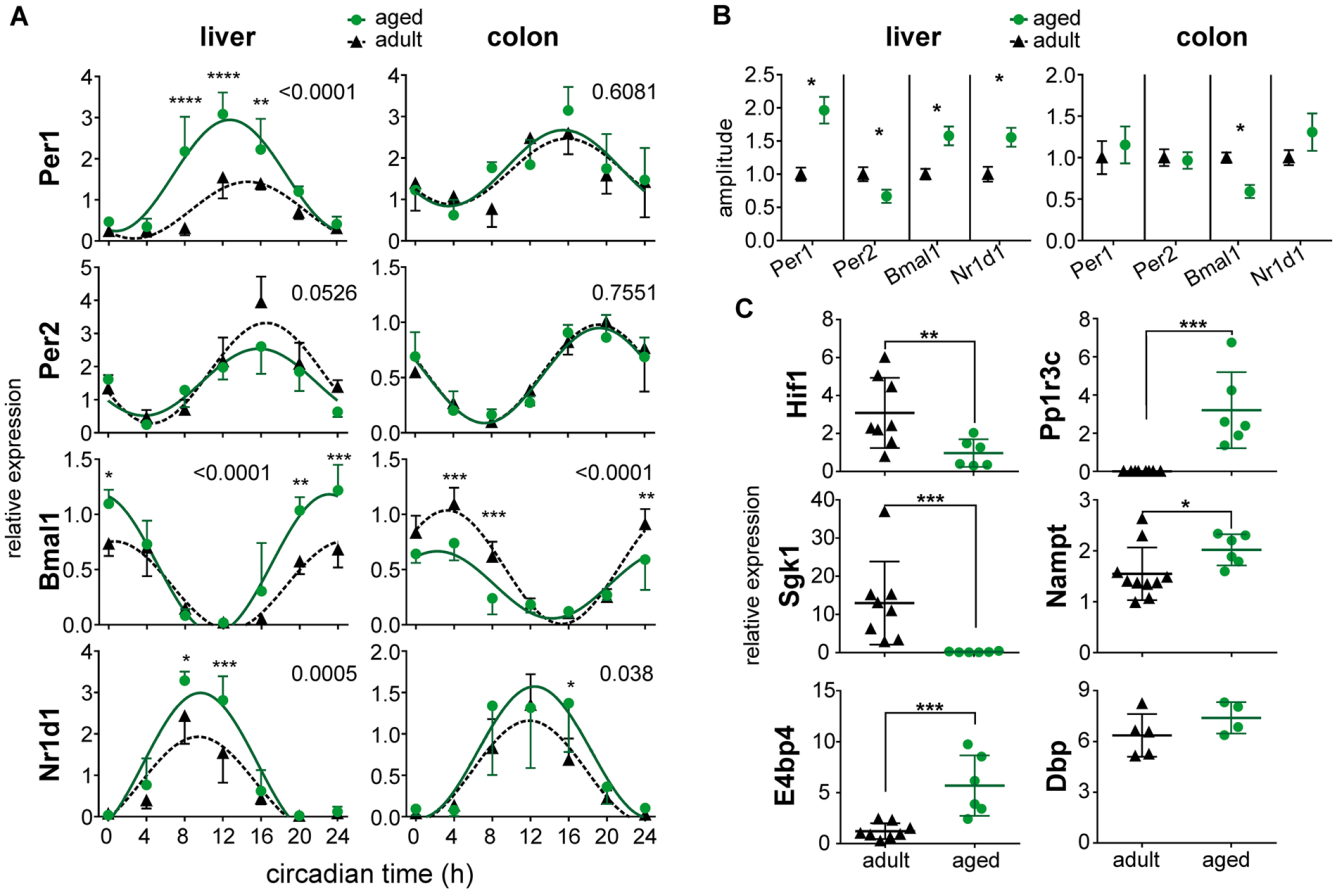
Effect of aging on expression of clock genes in the liver and colon *in vivo*. (**A**) Daily profiles of relative expression of *Per1*, *Per2*, *Bmal1* and *Nr1d1* were detected by RT qPCR in samples of adult (black triangles, dashed line) and aged (green circles, solid line) animals. Collection of the tissue samples was performed as described in Fig. 2. For each gene and tissue, the results of a 2-way ANOVA comparison between the profiles of adult and aged animals are depicted as the P value. The data were fitted with cosine curves. (**B**) Comparison of amplitudes of the cosine fits of rhythmic gene expression profiles in adult (black triangles, dashed line) and aged (green circles, solid line) animals depicted in A). Data are expressed as the mean ± SEM. (**C**) The expression in the liver of adult (black triangles, dashed line) and aged (green circles, solid line) animals; expression of *Hif1*, *Pp1r3c*, *Sgk1*, *Nampt*, and *E4bp4* was detected at circadian time 0 and expression of *Dbp* at circadian time 12. For each group, data are depicted as individual levels as well as the mean ± SD. *P < 0.05; **P < 0.01; ****P < 0.0001.

The daily expression profiles of the clock genes *Per1*, *Per2*, *Bmal1*, *Nr1d1* and *Clock* were compared between adult and aged animals (Fig. 2A). In the pancreatic tissue, aging had a significant and selective effect on the amplitude of the *Bmal1* expression profile (Fig. 2A,B), whereas it had no effect in the lungs (Fig. 2A,B). Interestingly, the amplitude of the *Bmal1* expression profile was also significantly elevated in the liver of the aged animals (Fig. 3A), i.e., in another glucose metabolism-relevant tissue, but not in the colon, where the amplitude was rather decreased in aged animals. In contrast, the *Clock* expression was significantly downregulated in both pancreas (P < 0.0001) and lungs (P = 0.0001) of aged animals (Fig. 2A,B). However, the number of BMAL1-immunopositive cells within the islets of Langerhans did not differ between adult and aged mice (Suppl. Figure 2).

Aging affected expression of some metabolism- and clock-related genes in the liver (Fig. 3B); namely, it suppressed expression of *hypoxia-inducible factor 1 Hif1a* (P = 0.008) and elevated expression of *protein phosphatase 1* (*Pp1r3c*) (P = 0.0007), *nicotinamide phosphoribosyltransferase* (*Nampt*) (P = 0.0312) and *nuclear factor* (*E4bp4*) (P = 0.0007) but did not affect *D-box binding protein* (*Dbp*) (P = 0.2857). Notably, aging completely suppressed expression of *serine/threonine protein kinase 1* (*Sgk1*) (P = 0.0007).

### Circadian clocks in pancreas and lungs *in vitro* differ in their resilience to aging

**Figure 4:**
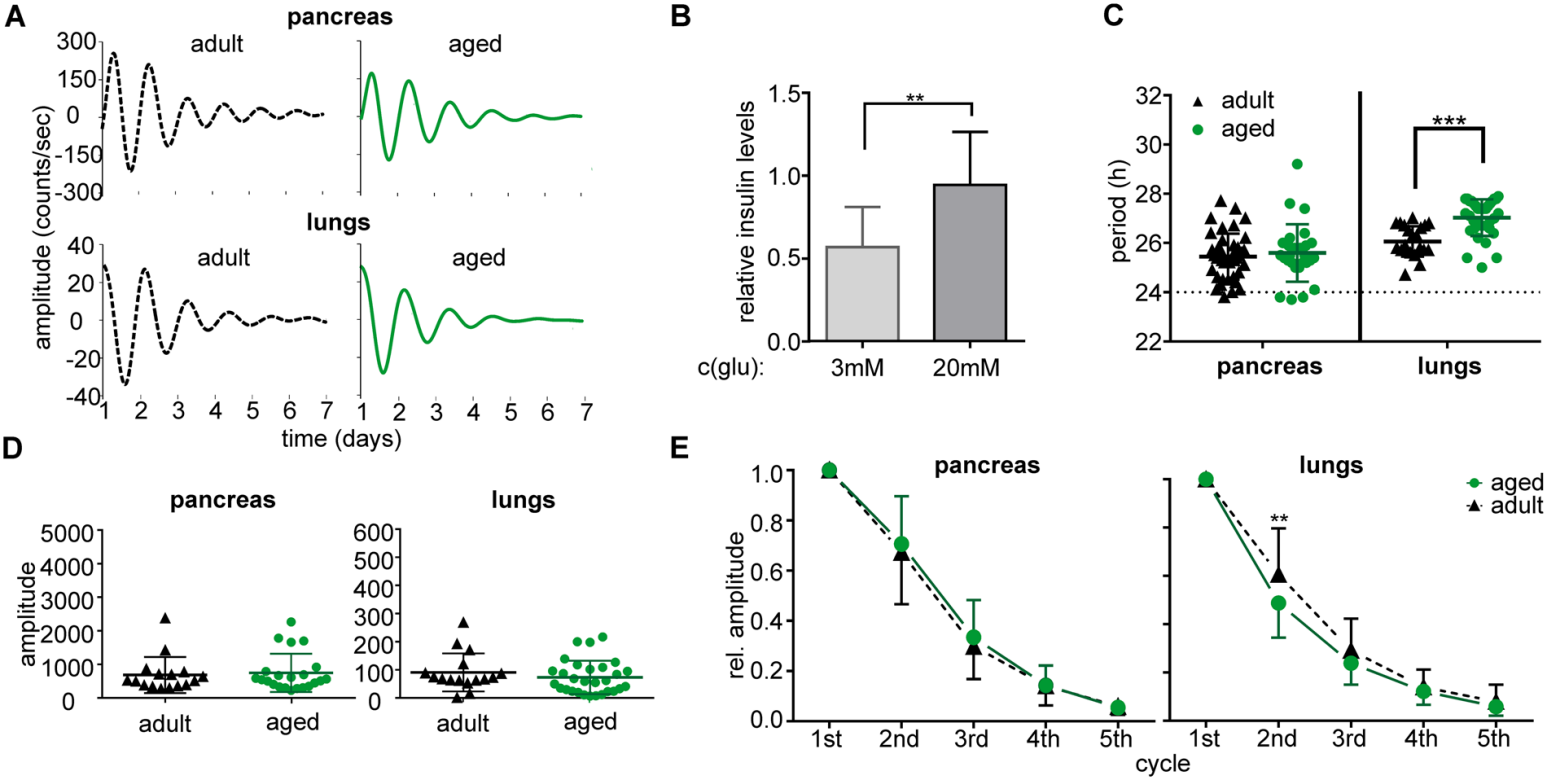
Effect of aging on properties of the circadian clocks *in vitro* in animals maintained in a light/dark regime (LD12:12). Bioluminescence rhythms were recorded from organotypic explants of pancreas and lungs prepared from *mPer2*^*Luc*^ adult (black triangles and/or dashed line) and aged (green circles and/or solid line) mice. (**A**) Representative bioluminescence rhythms of organotypic explants of pancreas and lungs recorded without changing the culture medium for 7 days. (**B**) Functional test of the pancreatic explant viability. The organotypic explants of the pancreas retained the ability to respond to glucose stimulation (from 3 mM to 20 mM glucose) by significant elevation of insulin release into the culture media *in vitro* (normalized to basal insulin secretion in medium with 11 mM glucose). Comparison of (**C**) periods and (**D**) amplitudes of the bioluminescence rhythms of organotypic explants of pancreas and lungs of adult (black triangles) and aged (green circles) mice. For each group, data are depicted as individual levels and mean ± SD. (**E**) Comparison of the amplitude dampening rate of the bioluminescence rhythms recorded from pancreas and lungs of adult (black triangles and/or dashed line) and aged (green circles and/or solid line) animals. Amplitudes of 5 subsequent cycles were detected and normalized to the amplitude of the first cycle. Data are expressed as the mean ± SD (n = 14–30). **P < 0.01; ***P < 0.001.

Representative traces of bioluminescence of pancreatic and lung explants from adult and aged mice maintained under LD12:12 are depicted in Fig. 4A. First, we determined that the organotypic explants of the pancreas retained their ability to respond to glucose application with a significant increase in insulin in the media (P = 0.0063) (Fig. 4B). Analyses of the bioluminescence rhythms (Fig. 4C–E) revealed tissue-specific differences: In the pancreas, aging did not affect the period (P = 0.6260), amplitude (P = 0.7245), or rate of the amplitude dampening (P = 0.4502) of the rhythm. In lungs, aging significantly lengthened the period (P = 0.0002), and although the amplitude of the first peak was not affected by aging (P = 0.081), the amplitude dampened faster (P = 0.0088) in aged mice. Altogether, in animals maintained under standard LD12:12 conditions, aging did not affect the robustness and persistence of the pancreatic clock *in vitro* whereas it had a negative impact on the clock in the lungs.

### LL affects properties of the circadian clock in the pancreas independently of age

Because the circadian clock in the pancreas was resilient to aging *in vitro*, we tested whether the exposure of animals to LL, which has deleterious effect on SCN output rhythms, affects its properties and whether that effect is influenced by aging.

**Figure 5:**
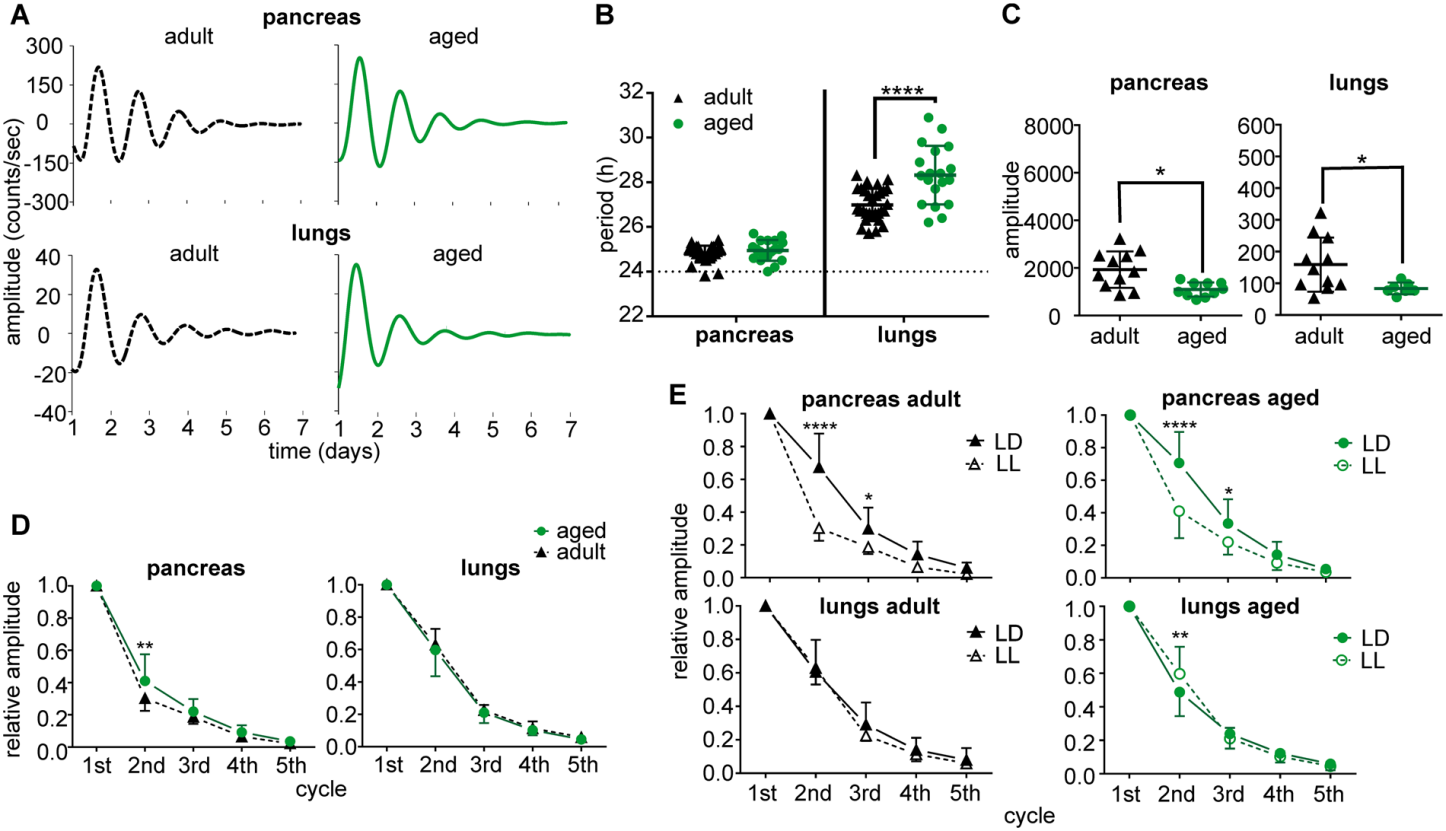
Effect of aging on properties of the circadian clocks *in vitro* in animals maintained in constant light (LL). Bioluminescence rhythms were recorded from organotypic explants of pancreas and lungs prepared from *mPer2*^*Luc*^ adult (black triangles and/or dashed line) and aged (green circles and/or solid line) mice. (**A**) Representative PER2-driven bioluminescence rhythms of organotypic explants of pancreas and lungs recorded without changing the culture medium for 7 days. Comparison of (**B**) periods and (**C**) amplitudes of the bioluminescence rhythms of organotypic explants of pancreas and lungs of adult (black triangles) and aged (green circles) mice. For each group, data are depicted as individual levels and mean ± SD. (**D**) Comparison of the amplitude dampening rate of the bioluminescence rhythms recorded from pancreas and lungs of adult (black triangles and/or dashed line) and aged (green circles and/or solid line) animals on LL. Amplitudes of 5 subsequent cycles were detected and normalized to the amplitude of the first cycle. Data are expressed as the mean ± SD (n = 7–11). (**E**) Comparison between amplitude dampening rates of the bioluminescence rhythms of explants from animals maintained in LD12:12 and LL; explants of adult animals on LD (filled black triangle) and LL (open black triangle), and explants of aged animals on LD (filled green circle) and LL (open green circles). Amplitudes of 5 subsequent cycles were detected and normalized to the amplitude of the first cycle. Data are expressed as the mean ± SD (n = 7–30). **P < 0.01; ****P < 0.0001.

Explants of pancreas and lungs from adult and aged animals maintained in LL exhibited circadian rhythms (Fig. 5A). For the pancreas, exposure to LL slightly shortened the mean circadian period when compared to LD12:12 both in adult (24.8 ± 0.4 h compared to 25.5 ± 0.9 h; respectively; P = 0.0108) and in aged (25.0 ± 0.5 h compared to 25.6 ± 1.2 h; respectively; P = 0.0359) mice. Aging had no effect additional to LL on the period length of the rhythm (P = 0.6260) (Fig. 5B); although it decreased the amplitude of the rhythm (Fig. 5C), it had rather a decelerating effect on the rate of its dampening (P = 0.0048) (Fig. 5D). In fact, exposure to LL had a much larger effect than aging, because the rhythms dampened significantly faster in the pancreas of adult as well as aged mice compared to LD12:12 conditions (Fig. 5E). In lungs, exposure to LL significantly lengthened the periods of the rhythms compared to LD12:12 in adult (27.0 ± 0.7 h compared to 26.1 ± 0.6 h; respectively; P = 0.0004) and aged (28.3 ± 1.3 h compared to 27.0 ± 0.7 h; respectively; P < 0.0001) animals. Aging thus had an additional effect on the LL-modulated period of the clock in the lungs in that it significantly further lengthened the LL-prolonged period (Fig. 5B) of the rhythm and suppressed its amplitude (Fig. 5C). However, the rate of the amplitude dampening in lung explants was affected neither by age (P = 0.2985) nor by exposure to LL (P = 0.2995) (Fig. 5D,E).

**Figure 6:**
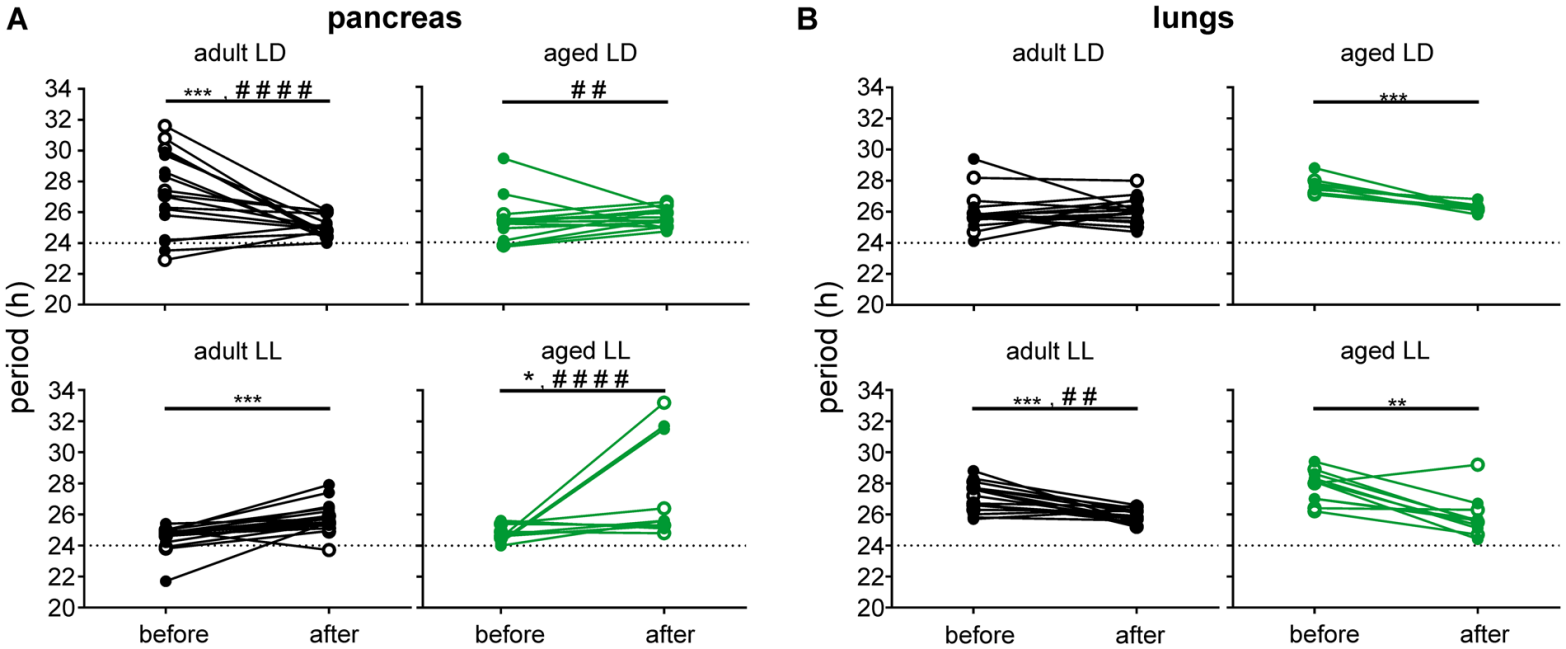
Effect of *in vitro* treatment on periods of organotypic explants of adult (depicted in black) and aged (depicted in green) animals maintained under light/dark (LD12:12) or constant light (LL) conditions. The periods were detected before and after the treatment performed during 5 days consisting of application of 1 µl solution followed by medium exchange (open and filled circles apply to different solutions preceding the medium exchange as described in Methods), and the effects of the treatment on the period were statistically evaluated. The resulting t-test P values for differences between the periods before and after the treatment are depicted by asterisks (*P < 0.05; **P < 0.01; ***P < 0.001), while the F test P values for differences between inter-individual period variances are depicted by hash marks (^##^P < 0.01; ^####^P < 0.0001).

The next question was whether LL exposure, which impaired coherence of the explants (Fig. 5E), affected the ability of these explants to be synchronized by rhythmic cues. A rhythmic signal was imposed on the explants via the *in vitro* treatment described in the Materials and Methods, and the treatment was considered to have entraining effect if it caused a significant change in the period lengths and/or a decrease in their inter-individual variability (Fig. 6). In pancreatic explants (Fig. 6A) of mice maintained in LD12:12, the treatment significantly decreased the mean period values and/or their individual variability in adult animals (periods before: 29.4 ± 6.3 h and after: 24.9 ± 0.7, P = 0.0006; variability: F test, P < 0.0001), as well as in aged animals (periods before: 25.3 ± 1.5 h, after: 25.6 ± 0.6 h; P = 0.4071; variability: F test, P = 0.0022). Importantly, after exposure to LL, the same treatment was not potent enough to entrain the clock in the pancreas. Although the mean period length had changed after the treatment in adult (period before: 24.5 ± 0.8 h, after: 25.8 ± 0.9 h, P = 0.0002) as well as aged (period before: 24.8 ± 0.6 h, after: 27.5 ± 3.3 h, P = 0.039) animals, its variability either did not change in adults (F test: P = 0.5825) or was even larger in aged animals (F test: P < 0.0001). For comparison, in lung explants (Fig. 6B) of animals maintained in LD12:12, the period did not change after the treatment in adults (before: 25.9 ± 0.8 h, after: 26.1 ± 0.9 h, P = 0.4297; variability: F test, P = 0.7631) but significantly shortened in aged (before 27.7 ± 0.5 h; after: 26.3 ± 0.3 h; P = 0.0003; variability: F test, P = 0.1212) animals. In LL, the treatment had a significant effect in explants from adult animals (period before: 27.1 ± 0.9 h; after: 25.9 ± 0.4 h, P = 0.0001; variability: F test: P = 0.0036) and a less robust effect in explants from aged animals (period before 27.8 ± 1.0 h; after: 25.8 ± 1.3 h, P = 0.0013; variability: F test, P = 0.4919). Notably, LL exposure significantly impaired the ability of the pancreatic, but not the lung, explants to be entrained by treatment *in vitro*.

## Discussion

Our results revealed that in aged (24–26 months old) *mPer2*^*Luc*^ mice the glucose homeostasis was significantly altered compared to adults (8–13 months old); their pancreas produced higher levels of insulin in spite of lower basal glucose levels; additionally, they had a lower rate of glucose clearance measured by IPGTT. The effect of aging on basal insulin levels has been controversial; both an increase^27^ and a decrease^28^ in insulin release have been reported. Additionally, age-dependent impairment in glucose clearance has been described^5,28,29^. The mechanism behind the elevated insulin levels in our aged mice might relate to a previously reported age-dependent increase in the ratio of large islets^3,4^, which have a higher insulin secretion rate^5^. It also could be due to a possible age-dependent decline in the insulin removal rate^30^, which could be due to a decline in insulin-degrading enzyme (IDE) levels in the liver^31^. In humans, the effect of aging on increase in insulin levels has not been confirmed after correction for visceral fat or diet^32–34^. However, in our study, significantly higher insulin levels were found in aged mice fed with the same diet as adult mice even after correction for their body weight. Higher insulin levels in our aged animals correlated with increased *Pclo* mRNA levels in the pancreas. Although we did not analyse levels of PCLO protein or activity, the functional relationship could be expected because *Pclo* is one of the crucial factors regulating pancreatic cAMP-dependent exocytosis and downregulation of its expression in β-cells significantly impaired insulin secretion^35^. Expression of *Pdx1*, a transcriptional factor involved in glucose- but not non-glucose-stimulated insulin secretion^36^, was also higher in our aged animals, as previously reported^37^. Expression of *Slc2a2*, a β-cell glucose transpoter GLUT2 required for normal glucose-stimulated insulin release, was lower in our aged animals, which may result in the decrease in pancreatic glucose sensitivity. The lower basal levels of plasma glucose we observed in aged animals were likely a consequence of increased glycogen storage in the liver as a compensatory mechanism to the higher insulin levels because we found significantly higher expression of *Pp1r3c*, which is a gene coding protein phosphatase 1, which promotes hepatic glycogen synthesis^38^. In line with this, expression of *Sgk1*, previously shown to influence glucose transport rate^39^, was dramatically suppressed in the liver of our aged animals. Therefore, downregulation of *Sgk1* expression may participate in the lower glucose uptake as well as in the lower rate of glucose clearance that we observed in aged mice by IPGTT. Additionally, in the liver of aged animals we detected significant changes in expression of metabolism-related genes (*Nampt*, *Hif1a* and *E4bp4*) in accordance with recently described aging-induced changes in the liver transcriptome^26^. Altogether, our aged animals exhibited signs of hyperinsulinemic hypoglycaemia that indicates changes in their pancreatic function and related glucose homeostasis.

These findings provided us with a rationale to study the effect of aging on the pancreatic clock. Comparing the daily profiles of clock gene expression in various peripheral tissues, we were unable to detect gross suppression of amplitudes of clock gene expression profiles due to aging in any of those studied tissues. Instead, we detected only selective gene- and tissue-specific downregulation, such as for the *Clock* expression in the pancreas and lungs and the *Bmal1* expression in the colon and lungs. Surprisingly, expression of some clock genes was rather upregulated in aging. Remarkably, both in the pancreas and liver (but not in the lungs), *Bmal1* was rhythmic with significantly higher amplitude. In accordance, previous study revealed gene specific up- and down-regulation due to aging in the liver^26^. The apparent age-related decrease in *Clock* expression we found in the pancreas did not seem to affect the overall quality of oscillations of other clock gene expression. This is possibly due to BMAL1 protein being the rate limiting partner in the transcription heterodimer^40^. The consequence of the effect of aging on *Bmal1* expression in the pancreatic tissue is not clear because it did not lead to a significant effect on clock-controlled gene expression. Nevertheless, the *Bmal1* gene, apart from its major role in the circadian timekeeping mechanism^15^, may have also a role beyond this function^41^ and thus participate in the age-dependent deterioration of the pancreatic physiology. In other tissues and cell types, such as in epidermal and muscle stem cells, circadian oscillations were also reported to remain robust during aging but to undergo extensive reprogramming^42^.

Because we found expressions of both clock genes coding the positive elements of the molecular feedback loop mechanism to be affected by aging, that is upregulated (*Bmal1*) and downregulated (*Clock*), we considered providing evidence for changes in properties of the *in vitro* dissected aged clock might answer the question whether clock-dependent or clock-independent pathways are involved in aging of the pancreas. Resilience of the clock to aging would favour an additional non-circadian role of *Bmal1* in the pancreas. To test this hypothesis, we used organotypic explants of the pancreas from *mPer2*^*Luc*^ mice that allowed us to monitor the circadian clock in real time for several weeks. The explants represent an integral tissue composed of all cell types, i.e., the endocrine and exocrine pancreas, but β-cells in the explants are viable as we confirmed by the ability of the explants to produce insulin upon glucose stimulation. Isolated β-cells are not a suitable model for this type of experiment because their much shorter viability would preclude the long-term recording necessary for the study of their clock properties.

When animals were maintained under LD12:12 conditions, aging had no effect on properties of the circadian clock in the pancreas *in vitro*; there was no effect on the period and dampening rate in clock-driven bioluminescence rhythms. This was in remarkable contrast to the clock in the lungs that became less persistent due to aging, because the rhythms ran with prolonged periods and dampened faster. An effect of aging on the clock in the lungs was reported earlier^24^, although lung explants of the aged *Per1-luc* rats completely lost their rhythmicity.

The high resilience to aging of the circadian clock in the pancreas led us to explore whether and how the clock is sensitive to a situation that compromises the rhythmic inputs from the central clock in the SCN, because exposure to an environmental LD cycle may facilitate rhythmicity of peripheral clocks via SCN-independent mechanisms^43^. Previously, we found that rhythmicity of the SCN *in vitro* could be disturbed by exposure of animals to LL^19^, which may uncouple the SCN from peripheral clocks and impair rhythmicity in clocks in the tissues of the gastrointestinal tract (liver, duodenum and colon)^44^. Here, we provide evidence that exposure of mice to LL changes properties of the clock more robustly in the pancreas than in the lungs. In the lungs, the LL-induced lengthening in periods of the bioluminescence rhythms was fully reversible by *in vitro* daily treatment (simulating the regular rhythmic stimuli from the central clock), demonstrating a high sensitivity of the clock in the lungs to systemic rhythmic signals. In contrast, in the pancreatic explants, exposure to LL accelerated dampening of the clock-driven bioluminescence rhythms, and the LL-induced effect on their periods was not reversible by the treatment. Therefore, the pancreatic clock properties were changed due to the previous exposure to disturbed lighting conditions, rather than simply due to an absence of rhythmic signals.

In conclusion, from the comparison between properties of the pancreatic clock with the clock in a tissue not involved in glucose homeostasis (lungs), we provide evidence for apparently high resilience to aging of the core molecular clock mechanism in the pancreas in animals maintained under standard lighting conditions. We also demonstrate a significantly higher sensitivity of the pancreatic clock to disturbances in external lighting conditions that might provide a basis for previously demonstrated consequences of disturbances in the circadian system on disordered glucose homeostasis (including T2DM) in human subjects exposed to shift work^18^.

## Methods

### Experimental animals and procedures

Male and female *mPer2*^*Luc*^ mice (strain B6.129S6-*Per2*^*tm1Jt*^/J, JAX, USA)^45^ and Wistar rats (Czech Republic and Institute of Physiology, Czech Academy of Sciences) were housed individually in a temperature-controlled (21 ± 2 °C) animal facility and had free access to food and water throughout the experiment. They were maintained under a light/dark cycle with 12 h of light and 12 h of darkness (LD12:12), with lights (150–300 lx, depending on cage position) on between 06:00 and 18:00. Time is expressed as circadian time, i.e., time 0 corresponds to lights on, and time 12 corresponds to lights off during the LD regime. For constant light (LL), the mice were exposed continuously to light of the same intensity as during the LD12:12 cycle for up to 9 months. At the time of sampling, *mPer2*^*Luc*^ mice up to the age of 8–13 months were designated “adult”, and mice 24–26 months old were designated “aged”. For rats, the group of “adult” animals was 6 months old and that of “aged” animals was 22–26 months old. The daily profiles in the expression of clock gene *Bmal1* detected in the pancreas of adult Wistar rats and *mPer2*^*Luc*^ mice did not reveal any significant inter-species differences (Suppl. Figure 1) and, therefore, the results of both animal strains were involved in the study.

The Animal Care and Use Committee of the Institute of Physiology, in agreement with the Animal Protection Law of the Czech Republic as well as European Community Council directives 86/609/EEC, approved all experiments. All efforts were made to alleviate the suffering of the animals.

### Detection of *BMAL1* protein in the pancreas by immunohistochemistry

12-μm-thick sections of *mPer2*^*Luc*^ mouse pancreas were cut, mounted on slides, fixed in 4% paraformaldehyde in PBS and processed for immunohistochemistry using the standard avidin-biotin method with diaminobenzidine as the chromogen (Vector Laboratories, Peterborough, UK) as previously described^46^. For more details, see Suppl. Figure 2.

### Detection of gene expression *in vivo* using real-time RT qPCR

For detection of gene expression daily profiles, rats were released into constant darkness, and starting at circadian time 0, they were sacrificed under deep anaesthesia (thiopental, 50 mg/kg b.w., i.p.) every 4 h during a 24-h cycle. At each time point, samples of liver, lung, pancreas and colon were collected from 5 rats. The pancreatic tissue was homogenized using ceramic beads (1.4 mm, MoBio Laboratories, Carlsbad, CA, USA; MagNa Lyser, Roche, Basel, Switzerland) in isolation buffer with β-mercaptoethanol according to manufacturer’s instructions (GenElute Mammalian Total RNA Miniprep Kit; Sigma-Aldrich, St. Louis, MO, USA) immediately after tissue dissection. The method of sampling of other tissues was described previously^46^.

The RNA isolation and real-time RT qPCR were performed as previously described^46^. For specific primers, see Table 1. The ΔΔCt method was used for the quantification of relative cDNA concentration. Relative expression was achieved by normalizing the expression to the mean relative expression of two housekeeping genes; for colon, lungs and liver these were *β2-microglobulin (B2m)* and *glyceraldehyde 3-phosphate dehydrogenase (Gapdh)*, and for the pancreas, they were *TATA binding protein (Tbp)* and *ribosomal protein S18 (Rps18)*. Expression of these reference genes has been proven to be stable throughout the 24 h cycle^47^. For each tissue, samples from adult and aged animals were assayed in the same real-time RT qPCR run.

**Table 1:** List of primers.

primer - RefSeq	sequence
Gapdh - NM_017008	Forward: 5′-TGATTCTACCACGGCAAG-3′ Reverse: 5′-TGATGGGTTTCCCATTGAT-3′
Rps18- NM_213557.1	Forward: 5′-ACTGCCATTAAGGGTGTG-3′ Reverse: 5′-GTCAGGGATCTTGTATTGTC-3′
Pclo - NM_020098.1	Forward: 5′-GATCGATTCAGAAGAAAGATG-3′ Reverse: 5′-CCCAGATTCAAGCTAAAACG-3′
Pdx1 - NM_022852.3	Forward: 5′-TCATCTCCCTTTCCCGTGGAT-3′ Reverse: 5′-TATTCTCCTCCGGTTCTGCTG-3′
Dbp - NM_012543	Forward: 5′-GCTAATGACCTTTGAACCTG-3′ Reverse: 5′-AGTACTTCTCATCCTTCTGTTC-3′
Nampt - NM_177928	Forward: 5′-CTTTGGTTCTGGTGGCGCTTTGCTAC-3′ Reverse: 5′-GCCGGCCCTTTTTCGACCTTTTGTT-3′
Hif1a - NM_024359	Forward: 5′-AGCGGCTGGGGACACGAT-3′ Reverse: 5′-TGGCTTTGGAGTTTCAGAGGCAGGTAA-3′
Sgk1- NM_001193569.1	Forward: 5′-AAGAAGATCACGCCCCCATTTA-3′ Reverse: 5′-AGGCTTCCGCGGCTTTCAC-3
PP1r3c - NW_047565	Forward: 5′-CCGCTAAGTGCGTGGTGCGA-3′ Reverse: 5′-GGGGTGGTGAATGTGCCAAGCA-3′
E4bp4 - NM_053727	Forward: 5′-ACCGTGTGAAGGGCGTGGAT-3′ Reverse: 5′-GTGGCGGTGGGGGTGCTC-3′

### Intraperitoneal glucose tolerance test (IPGTT)

IPGTT was performed in *mPer2*^*Luc*^ mice maintained in LD12:12, as previously described^48^. Mice were fasted for 3 h before the IPGTT, and testing started at 12:00. Blood glucose was measured by glucose meter (GlucoLab, Infopia Co., Ltd., Korea) before injection (basal) and then 15, 30, 60, 120 and 180 min after glucose injection (2 g/kg b.w., i.p.). The incremental area under curve (AUC) was calculated^49^. For detection of basal insulin levels, blood was drawn after 3 h fasting before the glucose injection, and insulin levels in plasma were assessed by ELISA (BioVendor Brno, Czech Republic).

### Tissue explant preparation and bioluminescence monitoring

The *mPer2*^*Luc*^ mice were maintained in LD12:12 or LL and were sacrificed between 12:00 and 13:00 via rapid cervical dislocation under isoflurane anaesthesia; their lung and pancreas were removed, rinsed in HBSS buffer (Sigma-Aldrich), and individual explants were placed onto Millicell Culture Inserts (Merck, Darmstadt, Germany) inside 35-mm Petri dishes with 1 ml of air-buffered recording medium supplemented with 100 U/ml penicillin, 100 µg/ml streptomycin, 10 µg/ml gentamicin, 1x GlutaMAX (Thermo Fisher, Waltham, MA, USA), 10% foetal calf serum (Sigma-Aldrich) and 0.1 mM D-Luciferin (Biosynth, Staad, Switzerland). Pancreatic explants were pre-incubated for 1.5 h in 1 ml air-buffered recording medium and then placed onto fresh recording medium. The bioluminescence was recorded in a LumiCycle apparatus (Actimetrics, Wilmette, IL, USA) beginning immediately after setting the explant cultures. The raw bioluminescence traces were analyzed using LumiCycle Analysis software (Actimetrics). The data were baseline-subtracted using the 24-h running average and smoothed prior to fitting a damped sine wave to calculate the period and amplitude before and after the treatments.

To measure the dampening rate of the amplitude of the bioluminescence rhythms, the explants were left untreated for at least 5 days. The amplitudes of the subsequent peaks were expressed relative to the 1^st^ peak. The *in vitro* test of entrainment of the circadian clock was performed in another group of explants according to a previously published procedure^19^: the rhythms were recorded for the first 4 days after setting the *in vitro* culture, and then, the explants were exposed to a treatment that involved addition of 1 µl solution [1% ethanol/saline phosphate buffer with or without melatonin (Sigma-Aldrich); final melatonin concentration in the recording medium was 1 µM] at 08:00 and followed by fresh medium replacement 8 h later. The treatment was originally designed to test the effect of 8-h melatonin exposure on the explants. However, after completion of the experiment, it appeared that the effect of treatment on the period length was independent of the treatment with 1 µl solution (with or without melatonin) preceding medium exchange. The medium exchange itself had a strong effect whereas application of the solution without media exchange had no effect on the period length. Therefore, the effect of the treatment could be attributed solely to the medium exchange, and composition of the solution was not considered in the final analyses of the data. This treatment was performed every day on 5 subsequent days, and then, the explants were left in the recording medium for another 5 days. Only the explants that survived the treatment (exhibited measurable oscillations after the treatment) were considered for subsequent statistical analyses (approximately 90%) (Suppl. Figure 3).

### Insulin secretion from pancreatic explants

To test the viability of the islets of Langerhans within the organotypic explants of the pancreas from *mPer2*^*Luc*^ mice during the *in vitro* incubation, we used a modified glucose- stimulated insulin secretion (GSIS) protocol. Explants were cultivated in air-buffered recording medium (as described above) that was enriched with 11 mM glucose. After 5 days of cultivation, the concentration of glucose was decreased to 3 mM for 24 h and then increased to 20 mM to stimulate insulin secretion. Insulin concentrations in media were assessed by ELISA (BioVendor).

### Statistical analysis

Differences between daily gene expression profiles were analyzed by two-way ANOVA followed by the Holm-Sidak post hoc multiple-comparison test. The amplitudes of these profiles were determined using cosine analysis as described elsewhere^50^; for comparison, the amplitudes were normalized relative to the value of the adult group and compared by multiple t-tests with the Holm-Sidak correction, where applicable.

Differences between means in (i) the periods of the bioluminescence rhythms *in vitro* (ii) the plasma glucose and insulin levels, (iii) the insulin levels in media after GSIS, and (iv) the metabolic-relevant gene expression were compared using Student’s t-test with Welch’s correction; when the data did not show a normal distribution (according to the D’Agostino-Pearson omnibus normality test), the Mann-Whitney test was used. The F test was used to assess significant differences in the periods of bioluminescence rhythms before and after the treatment. P < 0.05 (adjusted for multiple comparisons when relevant) was set as the threshold of significance for all statistical tests. Statistical analyses were implemented in Prism 6 software (GraphPad, Inc., La Jolla, CA, USA).

## Electronic supplementary material

Supplementary Information
