# Supplementary material for: Alteration in glucose homeostasis and persistence of the pancreatic clock in aged mPer2Luc mice
Source: Sci Rep. 2018 Aug 3;8:11668. doi: 10.1038/s41598-018-30225-y (PMC6076295; doi:10.1038/s41598-018-30225-y)
Supplement: Supplementary file 1 — Supplementary Information [file 41598_2018_30225_MOESM1_ESM.pdf]

Supplementary information

Alteration in glucose homeostasis and persistence of the pancreatic clock in aged *mPer2^{Luc}* mice

Zuzana Novosadová[1,2], Lenka Polidarová[1], Martin Sládek[1], Alena Sumová[1]*

[1]Department of Neurohumoral Regulations, Institute of Physiology, the Czech Academy of Sciences, Prague, Czech Republic

[2]Faculty of Sciences, Charles University, Czech Republic

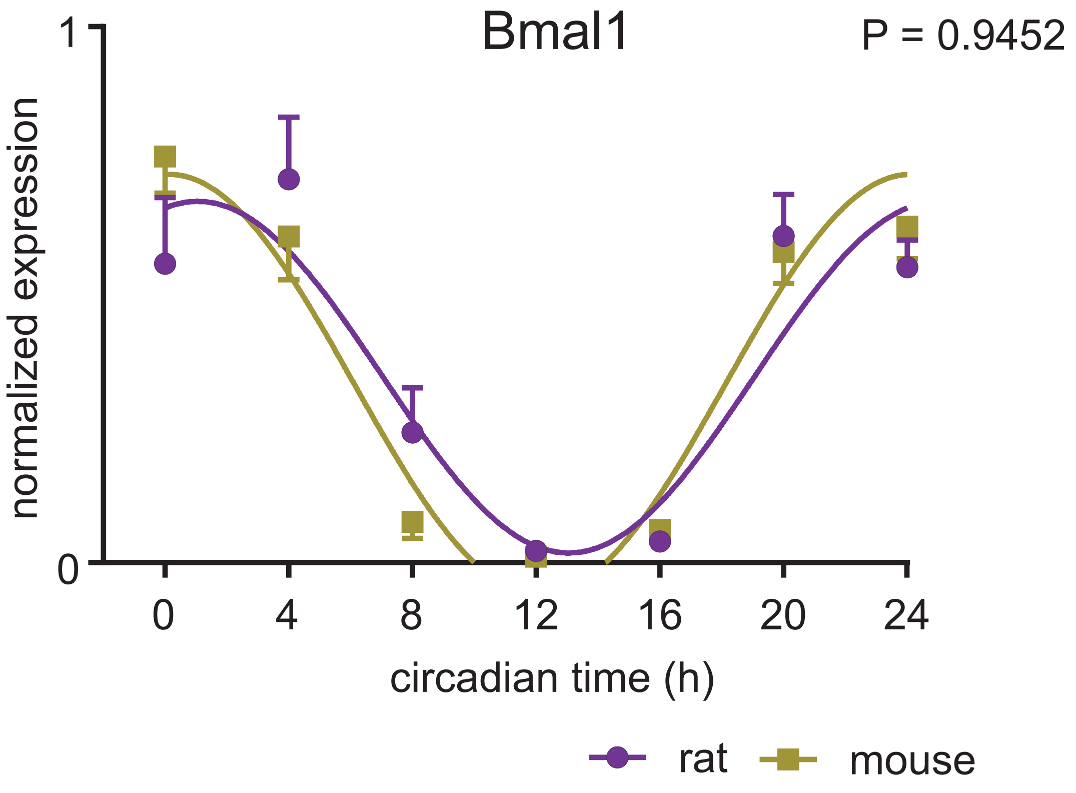

Supplementary Figure 1: A comparison of daily profiles of *Bmal1* expression in the mouse and rat pancreas detected by RT qPCR. The samples were collected every 4 h during the 24 h profile. Animals were sacrificed in darkness; circadian time 0 corresponds to lights on of the previous LD12:12 regime. At each time point, 5 (occasionally 4) animals were sacrificed. Depicted as mean normalised values ± SD. Statistical comparison by 2-way ANOVA did not reveal significant differences between the profiles (P value depicted in the figure).

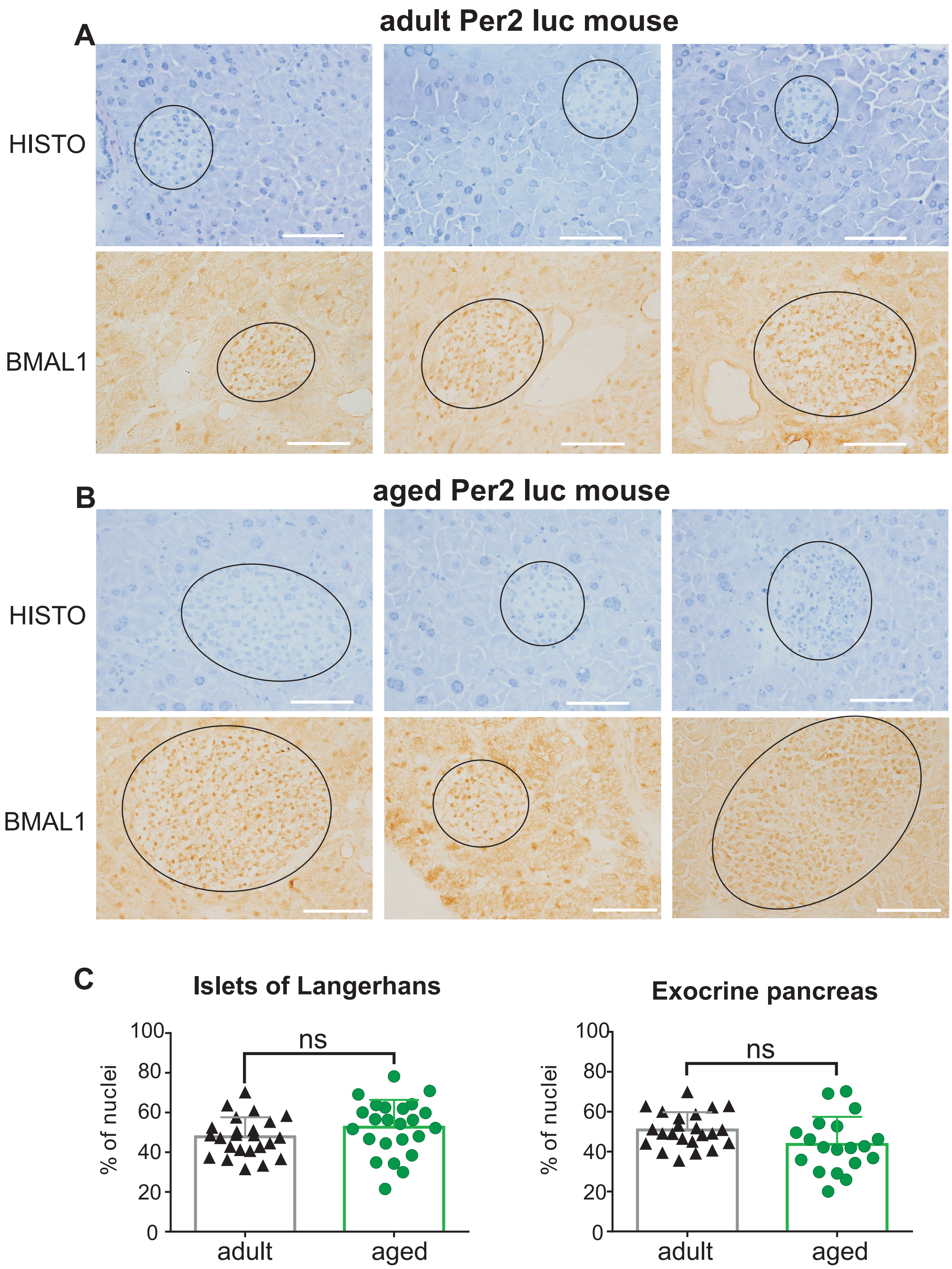

Supplementary Figure 2: Representative sections of snap frozen mouse pancreatic tissue of 3 young (**A**) and 3 aged (**B**) *mPer2*^{Luc} mice stained by Haematoxilin and Eosin (HISTO) and processed by immunohistochemistry (BMAL1). 12-µm-thick sections of *mPer2*^{Luc} mouse

pancreas were cut, mounted on slides, fixed in 4% paraformaldehyde in PBS and processed for immunohistochemistry using the standard avidin-biotin method with diaminobenzidine as the chromogen (Vector Laboratories, Peterborough, UK). The BMAL1 antibody was raised against the C-terminal 15 residues of mBMAL1 (GLGGPVDFSDLPWPL) using the Sigma-Aldrich custom peptide antibody service and was characterized previously [46]. As controls for background staining, parallel sections were treated simultaneously through the immunohistochemical procedure without incubation with the specific primary antibody. Islets of Langerhans are demarcated by black line in each picture. White bar corresponds to 100µm. **C)** The BMAL1 protein levels were quantified as the percent of immunopositive nuclei relative to the total number of nuclei as detected by DAPI fluorescence staining in each pancreas section, counted separately in the islets (left graph) and exocrine parts (right graph).

Reference:

46. Sládek, M. *et al.* Insight into the circadian clock within rat colonic epithelial cells. *Gastroenterology* **133,** 1240–9 (2007).

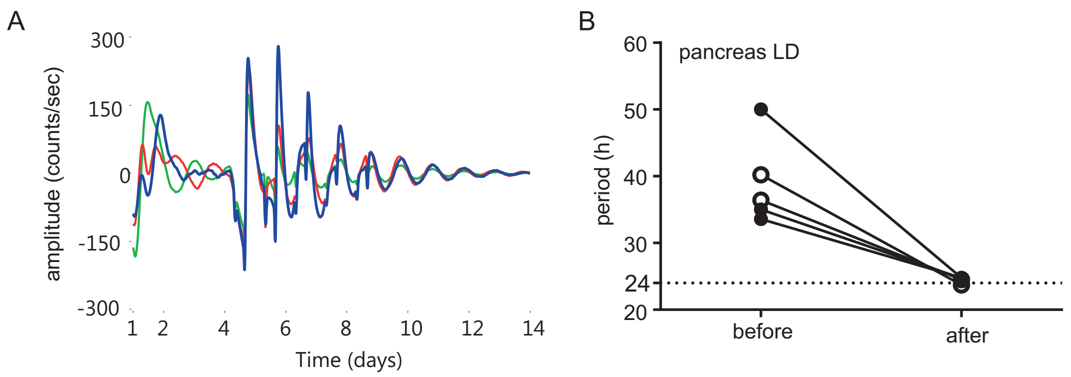

Supplementary Figure 3: A test of the viability of pancreatic organotypic explants of *mPer2*^{Luc} mice exhibiting periods outside the circadian range (more than 30 h) was provided by exposure of explants to the treatment procedure (described in detail in Methods section). A) Examples of traces of PER2-driven bioluminescence in pancreatic explants in vitro. B) Period of bioluminescence rhythms before and after the exposure of organotypic explants to the treatment procedure. This result confirmed that the long periods of the rhythm in the pancreatic explants were not due to their lower viability in culture conditions (B).
